# Loneliness During COVID‐19: An Overlooked Risk Factor for Post‐Pandemic Cognitive Decline in Older Adults

**DOI:** 10.1002/alz.087817

**Published:** 2025-01-09

**Authors:** Alaa Harb, Fernanda Carini da Silva, Maria Clara Ferreira de Jesus, Grace Willey, Juliana Nery Souza‐Talarico

**Affiliations:** ^1^ University of Iowa, Iowa City, IA USA; ^2^ University of Sao Paulo, São Paulo, São Paulo Brazil; ^3^ University of Sao Paulo, Sao Paulo, Sao Paulo Brazil

## Abstract

**Background:**

The COVID‐19 pandemic led to widespread social isolation and loneliness, especially among older adults aged 60 years and above. Loneliness is increasingly recognized as a significant public health concern given its association with adverse physical and mental health outcomes. However, less is known about the potential impact of loneliness on cognitive health and decline in older adults during the COVID‐19 pandemic specifically.

**Methods:**

A systematic review was conducted to examine the evidence on the relationship between loneliness and cognitive decline in older adults during the COVID‐19 pandemic. A comprehensive search was performed in MEDLINE (PubMed), CINAHL (EBSCO), PsycINFO (EBSCO), EMBASE, Scopus, AgeLine, and ProQuest for relevant studies published after January 2020 using PRISMA guidelines. Additional manual searches supplemented the database search. Inclusion criteria were studies with validated measurements of both cognitive function and COVID‐19 pandemic exposure in adults aged 60 years and older. Four paired reviewers independently assessed the identified studies for eligibility based on the predetermined criteria.

**Results:**

The search identified 9 eligible longitudinal studies with a total combined sample of 4,810 older adults. Three studies specifically measured loneliness using validated scales such as the UCLA Loneliness Scale. The most common assessment of cognition was the Mini‐Mental State Examination (MMSE), which was used in 8 of the 9 studies. Of the 4 studies examining the association between loneliness and cognitive decline, 3 reported a significant relationship even after adjusting for potential confounding factors including age, sex, and education level. However, the specific cognitive domains affected and magnitude of the association varied across studies.

**Conclusion:**

The available evidence from this systematic review suggests higher loneliness is significantly associated with cognitive decline in older adults during the COVID‐19 pandemic. This relationship remained even after accounting for demographic and socioeconomic characteristics. These findings highlight the need for post‐pandemic public health strategies that foster social engagement and interaction while maintaining appropriate physical distancing measures. Such approaches may help mitigate the secondary effects of the pandemic on mental and cognitive health among older adults. Further research is warranted to elucidate the complex interrelationships between social isolation, loneliness, and cognitive decline.

